# Supplementary material for: Differential regulation of the Epr3 receptor coordinates membrane-restricted rhizobial colonization of root nodule primordia
Source: Nat Commun. 2017 Feb 23;8:14534. doi: 10.1038/ncomms14534 (PMC5331223; doi:10.1038/ncomms14534)
Supplement: Supplementary Information — Supplementary Figures and Supplementary Tables [file ncomms14534-s1.pdf]

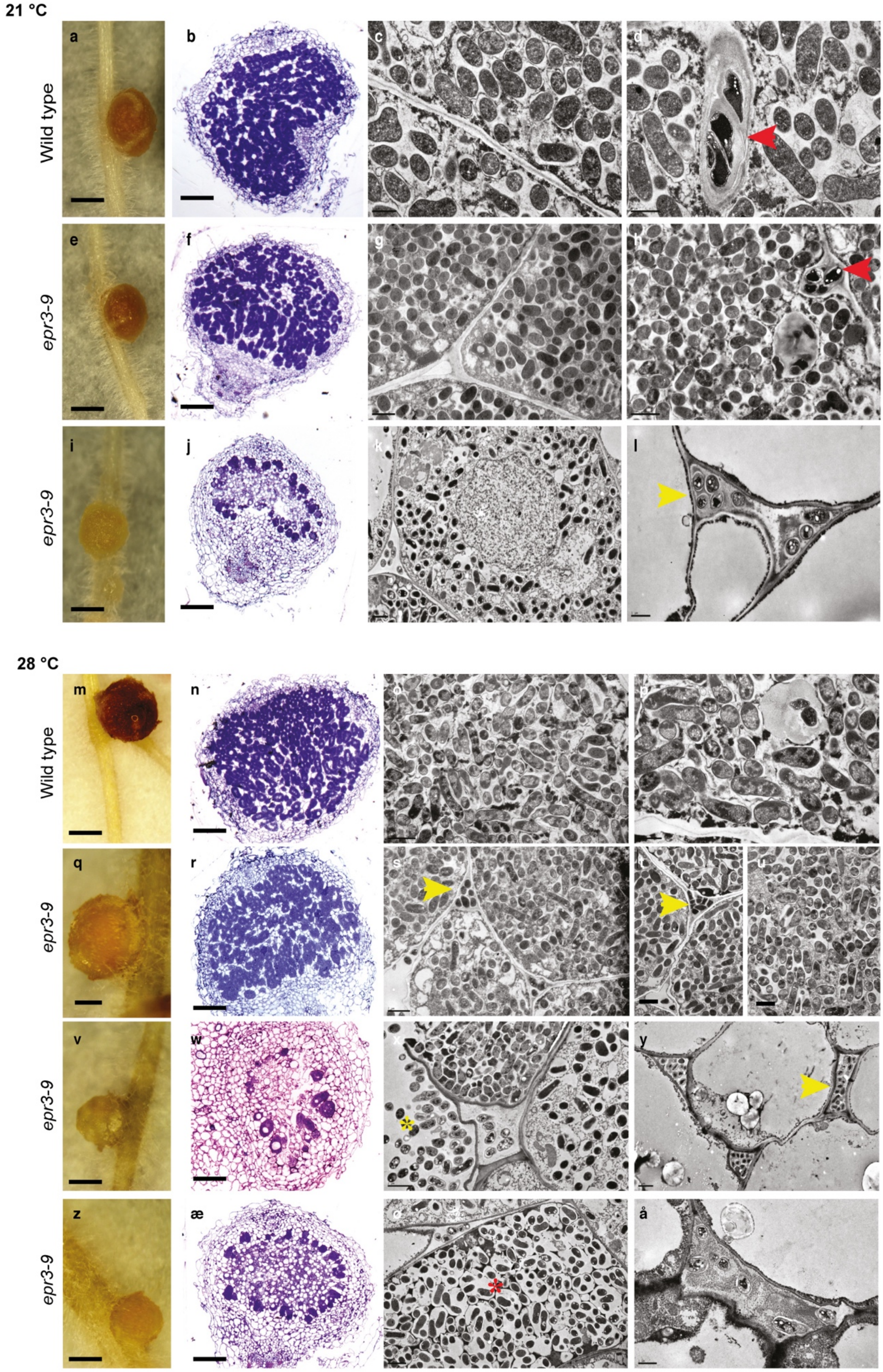

Supplementary Figure 1. Comparison of nodules from Gifu and *epr3-9* plants inoculated with *M. loti* MAFF303099 and incubated at 21 °C or 28 °C.

(**a** to **l**) are at 21 °C, and (**m** to **å**) are at 28°C. (**a**, **e**, **i**, **m**, **q**, **v**, **z**) Visual appearance of representative wild-type (Gifu) and *epr3-9* nodules (28 dpi). (**b**, **f**, **j**, **n**, **r**, **w**, **æ**) Light microscopy of the sectioned nodules shows the infection pattern. (**c**, **d**, **g**, **h**, **k**, **l**, **o**, **p**, **s**, **t**, **u**, **x**, **y**, **ø**, **å**) Transmission electron micrographs show the bacteroids enclosed in the symbiosome membrane and (**d**, **h**) infection threads (red arrows) containing rhizobia. (**l**, **s**, **t**, **y**) Nodules of *epr3-9* with infections between cells (yellow arrows). In *epr3-9* nodules, symbiosomes containing several bacteroids and infections between cells are frequently observed at 28°C. (**x**) Several cells appeared to be undergoing degradation (yellow star). (**Ø**)Several bacteroids enclosed within the same symbiosome membrane (red star). Scale bars: 500 µm (**a**, **e**, **i**, **m**, **q**, **v**, **z**), 200 µm (**b**, **f**, **j**, **n**, **r**, **w**, **æ**), 2 µm (**g**, **h**, **k**, **o**, **s**, **t**, **u**, **j**, **x**, **y**, **ø**), and 1 µm (**c**, **d**, **å**).

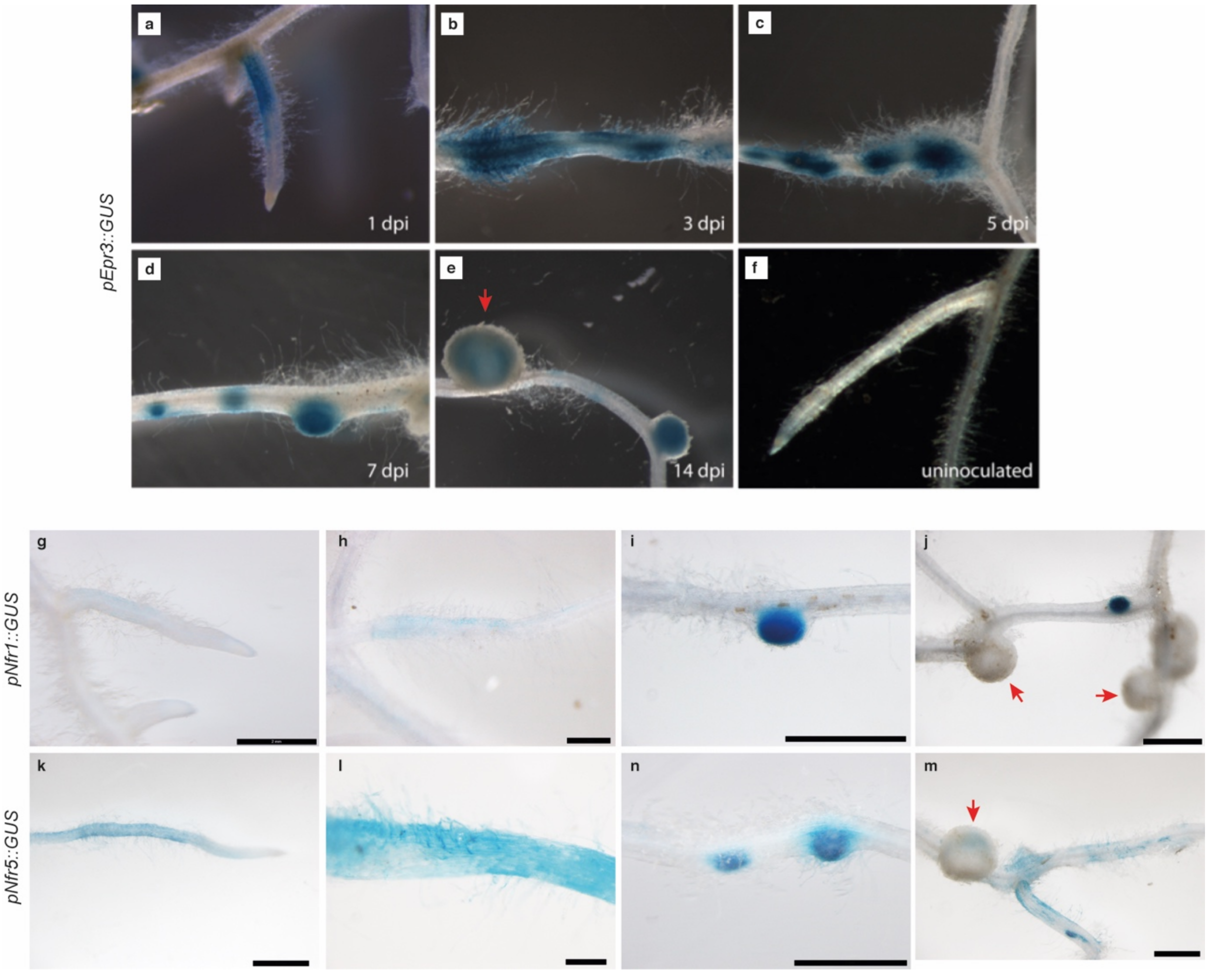

Supplementary Figure 2. ***pEpr3:GUS*****,** ***pNfr1:GUS*** **and** ***pNfr5:GUS*** **activity in Gifu in response to** ***M. loti*****.**

Wild-type plants transformed with the *pEpr3:GUS* reporter (**a**-**f**), *pNfr1:GUS* (**g**-**j**) and *pNfr5:GUS* (**k**-**m**) were inoculated with *M. loti* and stained for GUS activity at time points (**a**) 1 dpi, (**b**) 3 dpi, (**c**) 5 dpi, (**d**) 7 dpi and (**e**) 14 dpi. (**f**) Uninoculated *pEpr3:GUS* transformed wild-type showed no staining. (**h**, **i**, **j**, **l**, **n**, **m**) 7 dpi or 21 dpi. (**g**, **k**) Uninoculated roots expressing *pNfr1:GUS* and *pNfr5:GUS* are shown. *pEpr3:GUS*, *pNfr1:GUS* and *pNfr5:GUS* activity are lower and cease in older nodules (red arrows). Scale bars are 2 mm, except for **h**-1 mm and **l**- 0,5 mm.

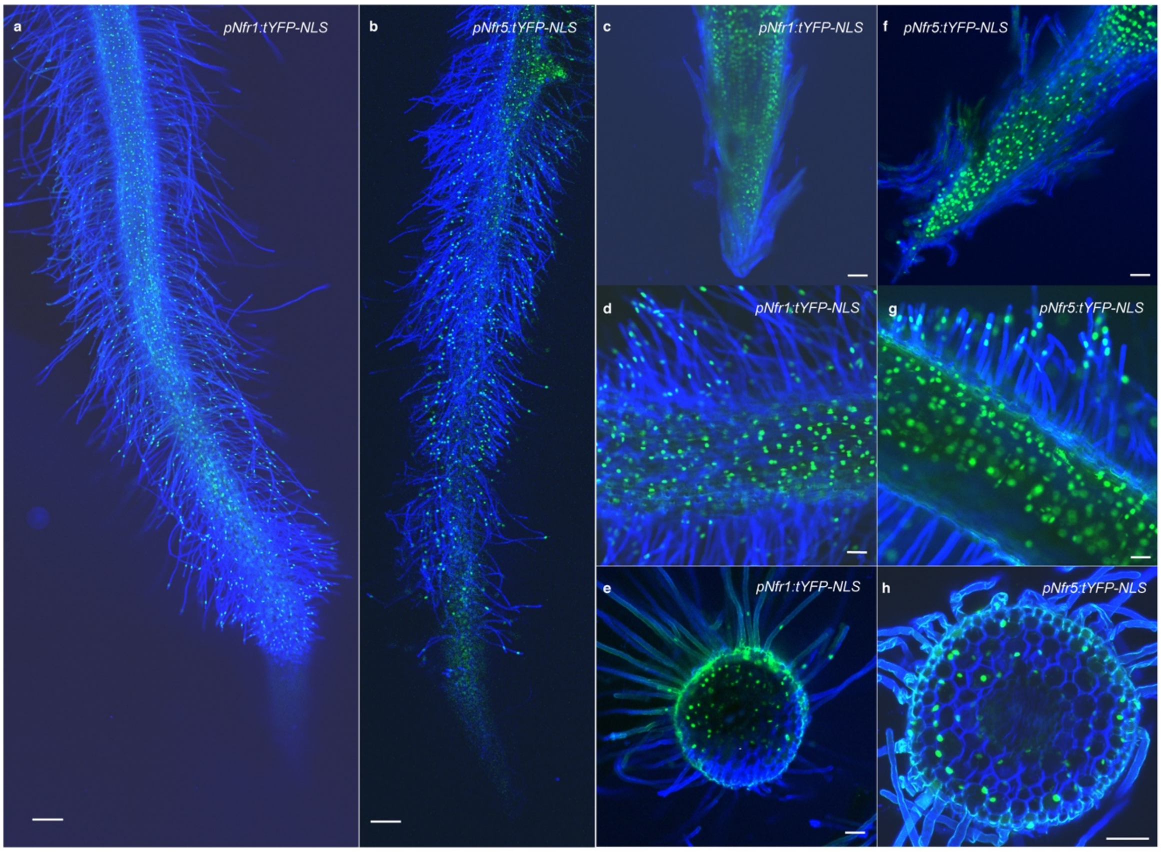

Supplementary Figure 3. *pNfr1:tYFP-NLS* and *pNfr5:tYFP-NLS* activity in uninoculated Gifu roots.
Gifu roots transformed with *pNfr1:tYFP-NLS* (**a**, **c**, **d**, **e**) and *pNfr5:tYFP-NLS* (**b**, **f**, **g**, **h**). **e** and **h** are cross sections. **a**, **b**; scale bars are 200 µm, **c**-**h**; scale bars are 50 µm.

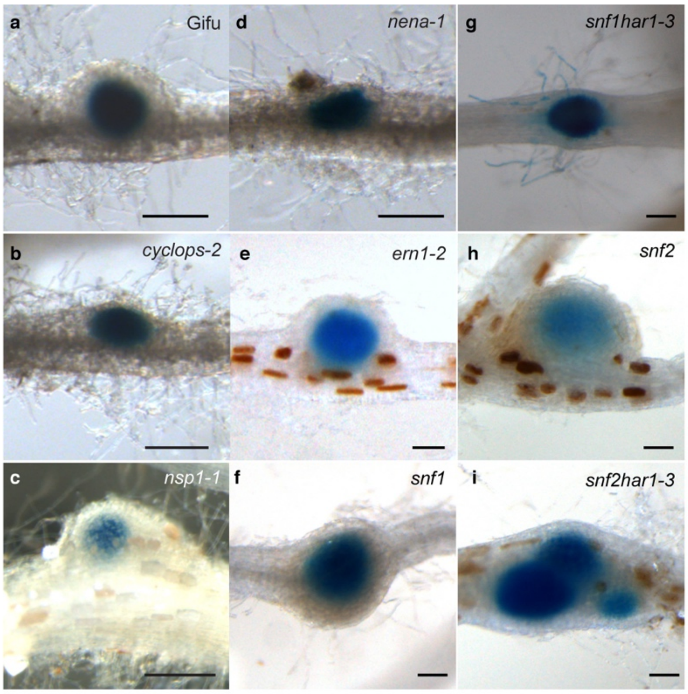

Supplementary Figure 4. Activity of *pEpr3:GUS* in nodule primordia of *Lotus* mutants. *Lotus* mutants transformed with *pEpr3:GUS*. (**a**) Gifu, (**b**) *cyclops-2*, (**c**) *nsp1-1*, (**d**) *nena-1*, (**e**) *ern1-2*, (**f**) *snf1*, (**g**) *snf1har1-3*, (**h**) *snf2* and (**i**) *snf2har1-3*. (**a** to **e**) are 10 to 21 dpi with R7A. (**f** to **i**) spontaneous nodules formed in the absence of rhizobia. Bars are 250 µm.

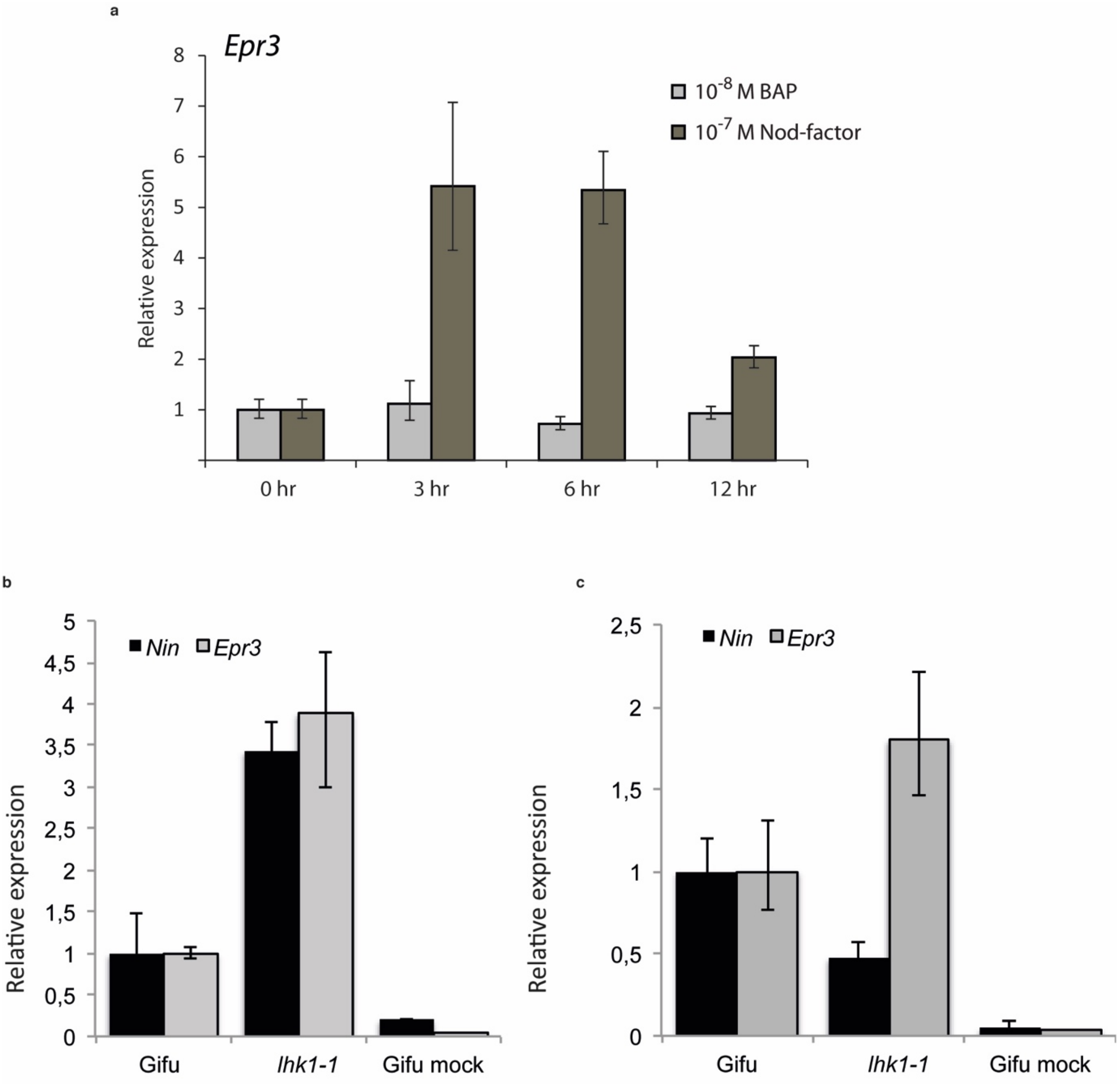

Supplementary Figure 5. Relative expression of *Epr3*

(**a**) The graph shows *Epr3* transcript levels measured by RT-qPCR in Gifu roots treated with 10^{-8} M BAP or 10^{-7} M Nod factor for 3 to 12 hours relative to 0 h/mock treatment. (**b**, **c**) Transcript levels measured by RT-qPCR at (**b**) 3 dpi and (**c**) 14 dpi. Bars show 95% confidence interval.

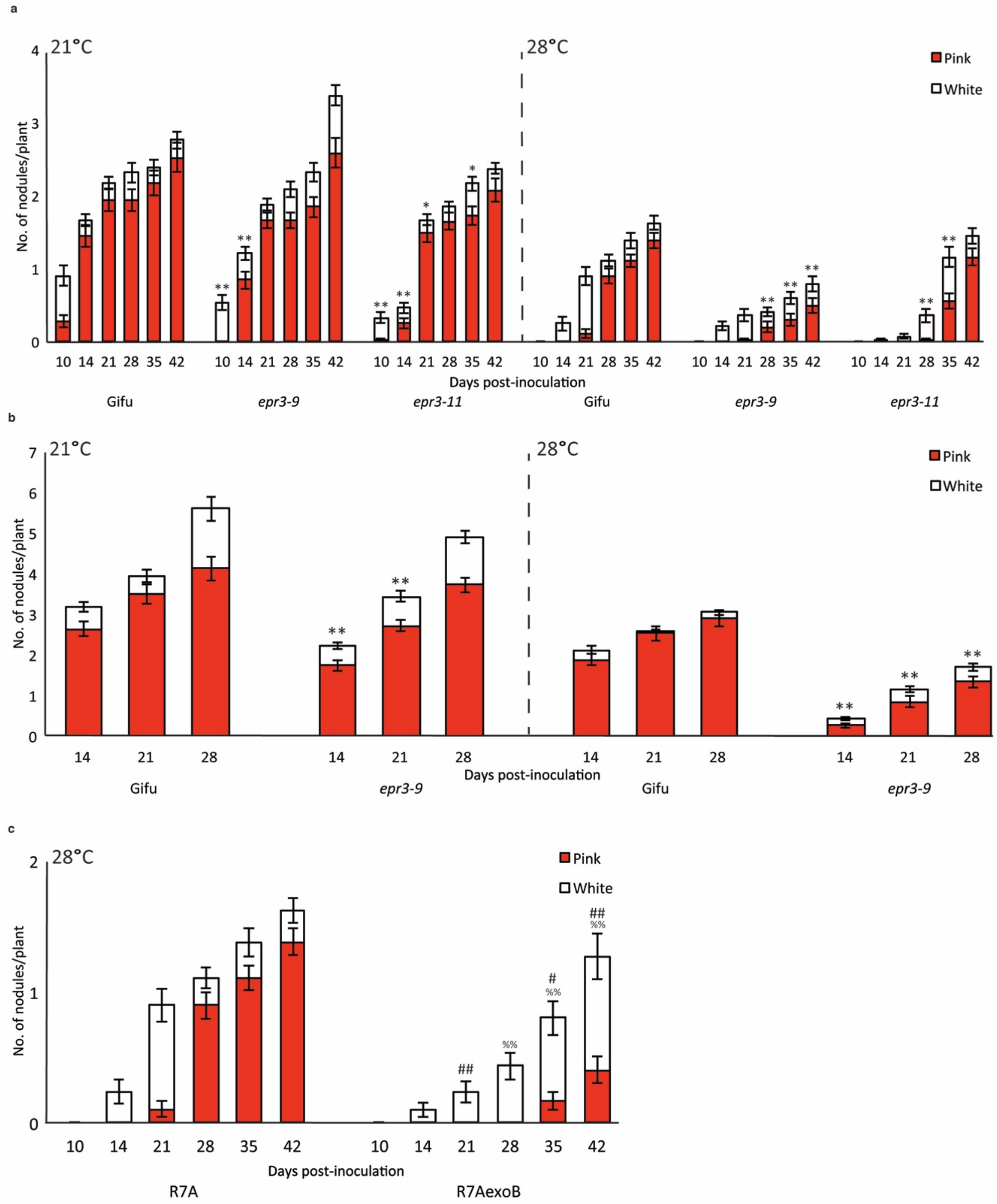

Supplementary Figure 6. Nodulation kinetics of Gifu and *epr3* mutants at 21 °C or 28 °C.

(**a**, **b**) Number of mature (pink) and immature (white) nodules formed by *L. japonicus* Gifu and *epr3-9* and *epr3-11* mutants after inoculation with *Mesorhizobium loti* R7A (**a**) or MAFF303099 (**b**), when grown at 21°C or 28°C. * ($p< 0.05$, student's t-test, n ≥ 29) and ** ($p< 0.01$, student's t-test, n ≥ 29) indicate significant differences in the number of mature pink nodules formed on *epr3* mutants compared to Gifu. (**c**) Nodulation kinetics of *L. japonicus* Gifu inoculated with R7A or R7AexoB and grown at 28 °C. # ($p< 0.05$, student's t-test, n ≥ 29) and ## ($p< 0.01$, student's t-test, n ≥ 29) indicate significant differences in the number of mature pink nodules, and % ($p< 0.05$, student's t-test, n ≥ 29) and %% ($p< 0.01$, student's t-test, n ≥ 29) indicate significant differences in the number of immature white

nodules formed on Gifu inoculated with R7AexoB compared to R7A. These error bars are SE.

Supplementary Table 1. ***Epr3*** **promoter activity in** ***Lotus*** **symbiotic mutants.**

Plant	n=	*M. loti* inoculation	*pEpr3:GUS* expression epidermal	*pEpr3:GUS* expression cortical	Nodule primordia
Gifu	58	+	+	+	+
Gifu	9	-	-	-	-
nfr1-1	15	+	-	-	-
nfr5-2	13	+	-	-	-
symRK-3	10	+	-	-	-
pollux-1	25	+	-	-	-/(+)**
nup133-3	20	+	-	-	-/(+)**
nena-1	16	+	-	+	-/(+)**
ccamk-13	19	+	-	-	-
cyclops-2	36	+	-/(+)*	+	+
cerberus-3	15	+	+	+	+
nsp1-1	33	+	+	+	+
nsp2-3	27	+	-	-	-
nin-2	30	+	+	-	-
ern1-2	54	+	-	+	+
nap-1	25	+	+	+	+
pir-1	10	+	+	+	+
lhk1-1	15	+	+	+	+
har1	17	+	+	+	+
snf1	30	-	-	+	+
snf2	15	-	-	+	+
snf1har1-3	10	-	+	+	+
snf2har1-3	7	-	-	+	+

* a very reduced expression
** temperature dependent for nodule formation

Supplementary Table 2. List of EMSA probes and primer sets

name	sequence (5' to 3')	tag at 5'
Nin 1	ATACCCTGCCCTTACAGACAAGTATAAGAGGCACAAAA	6-FAM™ at 5'
Nin 1 complement	TTTTGTGCCTCTTATACTTGTCTGTAAGGGCAGGGTAT	-
Nin 1 mutation	ATACCCTGCCTTTTCAGACAAGTACCCGAGGCACAAAA	6-FAM™ at 5'
Nin 1 mutation complement	TTTTGTGCCTCGGGTACTTGTCTGAAAAGGCAGGGTAT	-
Ern1	TGTCATTGTTATAGCCGTCTGAGATCCCAC	6-FAM™ at 5'
Ern1 complement	GTGGGATCTCAGACGGCTATAACAATGACA	-
Ern1 mutation	TGTCATTGTTATATTTGTCTGAGATCCCAC	6-FAM™ at 5'
Ern1 mutation complement	GTGGGATCTCAGACAAATATAACAATGACA	-

684 bp	fw (5' to 3')	CAACATAGATAAAAACAATTAG
	rev (5' to 3')	TTTCTTCAATGCAATGAGGAAG
329 bp	fw (5' to 3')	GAAGTGGTGGAACAGTCTGTG
	rev (5' to 3')	TTTCTTCAATGCAATGAGGAAG
280 bp	fw (5' to 3')	TTTGTCATTGTTATAGCCGTCTG
	rev (5' to 3')	TTTCTTCAATGCAATGAGGAAG
280 bp ERN1 mut (mut 1)	fw (5' to 3')	TTTGTCATTGTTATATTTGTCTG
	rev (5' to 3')	TTTCTTCAATGCAATGAGGAAG
280 bp NIN1 mut (mut 2)	fw (5' to 3')	TTTGTCATTGTTATAGCCGTCTGAGATCCCACAAAAGATACCCTGCCTTTTCAGACAAGTACCCGAGGCA
	rev (5' to 3')	TTTCTTCAATGCAATGAGGAAG
257 bp	fw (5' to 3')	AGATCCCACAAAAGATACCCTG
	rev (5' to 3')	TTTCTTCAATGCAATGAGGAAG
257 bp NIN1 mut	fw (5' to 3')	AGATCCCACAAAAGATACCCTGCCTTTTCAGACAAGTACCCGAGGCAC
	rev (5' to 3')	TTTCTTCAATGCAATGAGGAAG
Nsp2 gene	fw (5' to 3')	ATGGAAATGGATATAGATTGCATC
	rev (5' to 3')	CAGCTATGCACAATCTGATTCTG

All primers were added attB site for Gateway
